# Estimating cost-offsets of new medications: Use of new antipsychotics and mental health costs for schizophrenia

**DOI:** 10.1002/sim.4245

**Published:** 2011-04-26

**Authors:** A James O'Malley, R G Frank, S-L T Normand

**Affiliations:** a Department of Health Care Policy, Harvard Medical SchoolBoston, MA 02115-5899, U.S.A.; b National Bureau of Economic Research, Inc.Cambridge, MA, U.S.A.; c Department of Biostatistics, Harvard School of Public Health, BostonMA, U.S.A.

**Keywords:** bivariate likelihood, instrumental variables, medicaid cost data, method-of-moments, simultaneous equations, two-stage regression

## Abstract

Estimation of the effect of one treatment compared to another in the absence of randomization is a common problem in biostatistics. An increasingly popular approach involves instrumental variables—variables that are predictive of who received a treatment yet not directly predictive of the outcome. When treatment is binary, many estimators have been proposed: method-of-moments estimators using a two-stage least-squares procedure, generalized-method-of-moments estimators using two-stage predictor substitution or two-stage residual inclusion procedures, and likelihood-based latent variable approaches. The critical assumptions to the consistency of two-stage procedures and of the likelihood-based procedures differ. Because neither set of assumptions can be completely tested from the observed data alone, comparing the results from the different approaches is an important sensitivity analysis. We provide a general statistical framework for estimation of the casual effect of a binary treatment on a continuous outcome using simultaneous equations to specify models. A comparison of health care costs for adults with schizophrenia treated with newer atypical antipsychotics and those treated with conventional antipsychotic medications illustrates our methods. Surprisingly large differences in the results among the methods are investigated using a simulation study. Several new findings concerning the performance in terms of precision and robustness of each approach in different situations are obtained. We illustrate that in general supplemental information is needed to determine which analysis, if any, is trustworthy and reaffirm that comparing results from different approaches is a valuable sensitivity analysis. Copyright © 2011 John Wiley & Sons, Ltd.

## 1. Introduction

Estimation of the effect of one treatment compared to another in the absence of randomization is a common problem in biostatistics. With more emphasis placed on value in the health care setting illustrated with increased funding for comparative effectiveness research in the United States 1, researchers are increasingly utilizing observational studies to learn about effectiveness of interventions. It is well understood that a simple comparison of average outcomes between treatment arms will potentially confound the treatment effect with various selection effects (associations of predictors with treatment). If the treatment assignment mechanism depends on unmeasured variables affecting the outcome of interest (unmeasured confounders) then regression adjustment and propensity score methods 2 may fail to account for selection effects. In this case, instrumental variables methods may provide a pathway to causality. An instrumental variable (IV) is a random variable that is predictive of the treatment a patient receives but uncorrelated with the outcome conditional on treatment 3.

Despite the existence of the various estimators for IV analysis, there is little research on their comparative operating characteristics, and far less on empirical experience in real world settings. A compelling issue is that traditional instrumental variables methods are invariant to the form of the data (continuous versus binary outcome, continuous versus binary treatment) prompting the question of whether one can do better by tailoring methods to a given situation. Terza *et al.* 4 proposed a residual inclusion method for cases when the outcome or selection equation is nonlinear (e.g. as in generalized linear models). Another important consideration is that traditional IV methods do not utilize parametric assumptions, perhaps surprisingly so given the recent explosion in the adoption of latent variable models derived using parametric assumptions (e.g. IRT models, Rasch models, latent class models, latent factor models). An exception is the bivariate probit model, which is generated from assumptions on the underlying latent variables 5. We study the traditional IV methods, the residual inclusion method, and the latent variable approach to IV in the context of evaluating whether newer antipsychotic drugs are less costly than their predecessors. We focus on the estimation of the causal effect of a binary treatment on a continuous outcome.

Our research is motivated by the problem of comparing mental health spending between schizophrenia patients using newer *atypical* antipsychotic medications and those using older *conventional* antipsychotic medications in Florida's Medicaid population over the period 1994–2001. The older drugs, which are D2-antagonists such as chlorpromazine and haloperidol, were introduced in the 1950s to alleviate hallucinations and delusions in psychotic patients. Atypical antipsychotics, including clozapine, olanzapine (trade name zyprexa), quetiapine (trade name seroquel), and risperidone (trade name risperidal), were first marketed in the late 1980s and 1990s, and while considerably more expensive than the D2-antagonists, were associated with a different profile of side effects. While the conventional antipsychotics were associated with neurologic side effects, the newer atypicals have been linked to other side effects such as weight gain, diabetes, and lipid problems. During our study observation period, three atypicals were introduced—zyprexa, seroquel, and geodon. Some have claimed that atypical antipsychotics, while more expensive ultimately *pay for themselves* by leading to reductions in other types of health spending 6. This claim has come to be known as the offset hypothesis. The offset hypothesis asserts that the greater tolerability of the new antipsychotics will improve adherence to treatment regimens, thereby reducing relapses, resulting in declines in the use of hospital and emergency room services. However, it is disputed whether lower subsequent costs for atypicals are sufficiently large as to offset their greater upfront cost 7.

Study of the offsets hypothesis is complicated by the fact that patients that receive the newer atypical drugs likely differ from those getting the older drugs on a number of systematic factors that may not be fully measured. These include existing medical and mental health comorbidities, severity of illness, and treatment preferences.

We utilize variation in the availability of atypical drugs across the state of Florida that arises because the time-lag between Federal approval and local availability varies by geographic area to form instrumental variables. The instruments are indicators of whether a specific atypical was available in a patient's geographic area of residence defined as one of 11 area Medicaid offices representing geographic, cultural, social, and economic factors in a given year. Using these instruments we illustrate several different estimators that account for unmeasured selection effects to test the offsets hypothesis in the Florida Medicaid population. Our goal involves quantifying the evidence for or against the offsets hypothesis using multiple approaches encompassing different assumptions, thereby enabling one approach to act as a sensitivity analysis for another and yielding real-world experience of the extent to which methodological concerns about the various approaches matter. We also use simulations to evaluate the operating characteristics of the various methods when assumptions hold and when they are violated.

We next define notation, describe assumptions, and introduce models. General methods for estimation are detailed in Section 3 and implemented on the Florida Medicaid data in Section 4. Section 5 describes a simulation study to evaluate the operating characteristics of the methods when assumptions hold and when they are violated. We provide concluding remarks in Section 6.

## 2. Statistical models

### 2.1. Notation and definitions

We use simultaneous equations to specify models and the potential outcomes nomenclature of 8 to define treatment effects. Let *y*_*i*_, *z*_*i*_, ***x***_*i*_, ***u***_*i*_, and *c*_*i*_ denote the outcome, the treatment variable, a vector of exogenous covariates, a vector of instrumental variables, and an unmeasured confounding variable for the *i*th of *n* subjects.

The instrumental variables ***u***_*i*_ are assumed to be: (1) associated with *z*_*i*_ conditional on ***x***_*i*_, (2) uncorrelated with *y*_*i*_ conditional on (*z*_*i*_, *c*_*i*_, *x*_*i*_), and (3) uncorrelated with *c*_*i*_ conditional on ***x***_*i*_ 9, 10. Assumption (2) says that there is no direct effect of ***u***_*i*_ on *y*_*i*_ (the exclusion restriction), while assumption (3) says that ***u***_*i*_ shares no common causes with *y*_*i*_ (i.e. ***u***_*i*_ is uncorrelated with any unmeasured variables that predict *y*_*i*_). If assumption (3) is violated then ***u***_*i*_ may be related to *y*_*i*_ through an uncontrolled confounding variable 11, thereby introducing bias. In models where *y*_*i*_ is modeled with an explicit error term, ε_*y, i*_, assumptions 2 and 3 reduce to the assumption that ***u***_*i*_ and ε_*y, i*_ (which includes *c*_*i*_) are uncorrelated conditional on (*z*_*i*_, *x*_*i*_). Although ***x***_*i*_ and *c*_*i*_ might predict both *y*_*i*_ and *z*_*i*_ and so structurally are equivalent, *c*_*i*_ is problematic because it is unobserved. Controlling for ***x***_*i*_ generally makes assumptions (2) and (3) above more believable by controlling for variation in unmeasured confounders that is correlated with ***x***_*i*_ 12.

The annual mental health spending for patient *i*, denoted cost_*i*_, is the sum of all payments made for services with mental health diagnoses, mental health procedures (e.g. psychotherapy), or psychotropic drugs that are primarily used for mental health treatment such as antidepressants and mood stabilizers. The distribution of cost_*i*_ is right skewed. As discussed in Section 4.1, Box–Cox transformations under various models indicated that the log-transformation traditionally used for spending data to account for right-skew would be reasonable. Accordingly, *y*_*i*_ = log(cost_*i*_). Because all patients in the data set received services from a health care provider, the 29 observations with cost_*i*_ = 0 were considered impossible and excluded from the analysis.

The treatment *z*_*i*_ is a binary-valued indicator of whether a patient filled an atypical (*z*_*i*_ = 1) or a conventional antipsychotic (*z*_*i*_ = 0) prescription in a given year. If a patient filled both we assigned them to the drug that accounted for the greatest share of their health costs for that year. Thus, *z*_*i*_ is defined in the same year as cost_*i*_. In a sensitivity analysis we restricted the data to new users (i.e. those who initiated treatment with an antipsychotic during our study period) and the first year of data on each individual, thereby obtaining the subset of subjects for whom we could reasonably assume made their initial antipsychotic choice during the study period. This allowed us to check whether it made sense to combine new users and longer term users, and those staying on a single drug from those who switched drugs, in a single analysis. The results were minimally affected suggesting that a pooled analysis that controlled for year was justified.

The predictors in ***x***_*i*_ are race/ethnicity, female, age, receipt of Supplementary Security Income (SSI) benefits, history of substance abuse, area of residence, and year. Variables represented by *c*_*i*_ could include health status of the patient, access to skilled physicians, and physician prescribing habits. The vector of instrumental variables ***u***_*i*_ consists of the products of binary indicators of whether zyprexa, seroquel, and geodon were FDA-approved at the start of each year and the 10 area-of-residence indicators; the most populous area, Miami, was the excluded category. The variables (***x***_*i*_, *c*_*i*_, ***u***_*i*_) are all defined in the same year as cost_*i*_ and *z*_*i*_.

The rationale for the above choice for ***u***_*i*_ is that the availability of antipsychotics depends on physician learning which in turn depends on local area attitudes towards innovation, information dissemination, and other conditions that varied substantially across Florida. Thus, drug approval and area of residence are related to antipsychotic use at a given time. In order for ***u***_*i*_ to be an appropriate instrument, it cannot be directly related to health care costs or to unmeasured confounders affecting health care costs. This would not be the case if patients with higher costs lived in areas that were faster adopters or if attitudes towards innovation, information dissemination, and other conditions directly affect costs. Thus, the inclusion of area of residence indicator variables in ***x***_*i*_ helps make drug approval interacted with region a valid IV.

### 2.2. Assumed underlying model

The outcome *y*_*i*_ depends on treatment *z*_*i*_ and the exogenous predictors ***x***_*i*_ through the linear regression equation


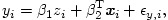
(1)

where ε_*y, i*_ has mean 0 and variance σ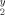. The validity of this model relies on the existence of linear relationships, homogeneous variances, independent observations, and orthogonality between (*z*_*i*_, ***x***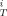)^T^ and ε_*y, i*_. In the Medicaid data, *z*_*i*_ and ε_*y, i*_ are likely to be correlated as (e.g.) detailed measures of the severity of a patient's health condition were not available, and these likely affect a patient's propensity to fill an atypical prescription and their net health spending.

A second equation describes the relationship between *z*_*i*_ and (***u***_*i*_, ***x***_*i*_)


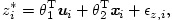
(2)

where 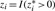. In terms of the Medicaid data, 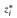 represents the patient's propensity to be prescribed an atypical antipsychotic. By assumption, the predictors on the right-hand side (rhs) of (2), including the IV ***u***_*i*_, are independent of ε_*y, i*_.

The regression parameter β_1_, the difference in the outcomes when all other factors (including those influencing ε_*y, i*_) are equal, is of primary interest. When *z*_*i*_ is exogenous (uncorrelated with ε_*y, i*_), β_1_ = *E*[*y*_*i*(1)_ − *y*_*i*(0)_∣***x***_*i*_], where *y*_*i*(*z*)_ denotes the potential outcome for subject *i* when *z*_*i*_ = *z*. However, if *z*_*i*_ is correlated with ε_*y, i*_ then β_1_ = *E*[*y*_*i*(1)_ − *y*_*i*(0)_∣***x***_*i*_, ε_*y, i*_]≢*E*[*y*_*i*(1)_ − *y*_*i*(0)_∣***x***_*i*_].

### 2.3. Parametric model: structural and distributional assumptions

In parametric analyses we follow the construction of the bivariate probit model. This model assumes that the error term ε_*i*_ = (ε_*y, i*_, ε_*z, i*_) is an additive function of *c*_*i*_, an unmeasured confounder that linearly affects 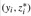, and (δ_*y, i*_, δ_*z, i*_), a random disturbance. That is, ε_*i*_ = (β_3_*c*_*i*_ + δ_*y, i*_, θ_3_*c*_*i*_ + δ_*z, i*_), where *c*_*i*_, δ_*y, i*_, and δ_*z, i*_ are mutually independent random variables each with mean 0 and variance σ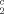, τ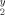, and τ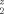, respectively. Hence, ε_*i*_ has mean **0** and covariance


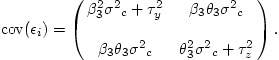


Because we can multiply σ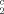 by *k*, and divide β_3_ and θ_3_ by *k*^1/2^ without changing the model, for model identification we set σ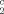 = 1.

Derivation of the bivariate probit is completed by assuming that *c*_*i*_, δ_*y, i*_, and δ_*z, i*_ are normally distributed, implying that ε_*i*_ is bivariate normal. Thus, the model is identified through the first and second moments of the distribution of ε_*i*_. Because *z*_*i*_ is binary we can only identify the standardized effects θ_1_/(θ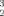 + τ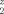)^1/2^ and θ_2_/(θ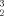 + τ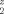)^1/2^, leading to the constraint θ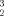 + τ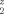 = 1. With three parameters and two degrees of freedom in cov (ε_*i*_) we set θ_3_ = 1 (equivalently, τ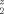 = 0) to identify the model, defining β_3_ and β_3_/(β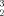 + τ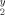)^1/2^ as the covariance and correlation between ε_*y, i*_ and ε_*z, i*_, respectively. In the normal case unobserved selection is thus quantified by ρ = β_3_/σ_*y*_, where σ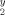 = β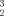 + τ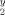. Clearly, ρ∈[−1, 1].

## 3. Estimation methods

### 3.1. Ordinary least squares (OLS)

Linear regression fits the model *y*_*i*_ = β_1_*z*_*i*_ + β
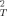
***x***_*i*_ + ε_*y, i*_ where var (ε_*y, i*_) = σ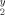. The least-squares estimator is 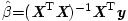, where ***y*** = (*y*_1_, …, *y*_*n*_)^T^ and ***X***is the *n* × *p* matrix with *i*th row (*z*_*i*_, ***x***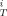). When ε_*y, i*_ is mean independent of all predictors and has homoscedastic variance (as assumed here), the estimator is minimum variance unbiased among the class of linear estimators (Gauss Markov theorem). However, if any predictor is correlated with ε_*y, i*_, OLS will be inconsistent 13, Chapter 5.

### 3.2. Two-stage least squares (2SLS)

The classic IV estimator of β in (1) is the minimum variance estimator among those satisfying the constraint that ***u***_*i*_ and ε_*i*_ are orthogonal (see Appendix A for construction). This method-of-moments estimator is equivalent to the two-stage least-squares (2SLS) procedure, in which we first fit


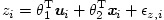
(3)

to obtain 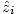, and then fit


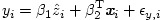
(4)

to estimate β. In the special case where ***u***_*i*_ is univariate-binary and there are no other covariates, the 2SLS procedure given by (3) and (4) is equivalent to the Wald estimator 14. The standard error of 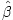 is


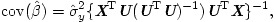
(5)

where ***U*** is the matrix with *i*th row (***u***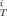, ***x***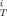) and 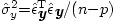 estimates the residual variance of the outcome equation 13, Section 5.2.2, 15, p. 531.

The orthogonality condition enforced in (3) holds factors affecting ε_*i*_ constant, allowing β to be estimated for those subjects for whom ***u***_*i*_ influences *z*_*i*_, the ‘population on the margin’. In the offsets analysis, the population on the margin is patients whose uptake of an atypical antipsychotic medication was influenced by the availability of zyprexa, seroquel, or geodon in the city where they lived. Thus 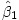 is a ‘structural shift’ of using an atypical.

A notable feature of 2SLS is that no presumption is made about the type (e.g. binary, ordinal, interval) of variables that *y*_*i*_ and *z*_*i*_ are or about the distribution of ε_*i*_. The binary nature of *z*_*i*_ led us to consider whether more efficient results could be obtained by accounting for the form of *z*_*i*_.

### 3.3. Alternative two-stage approaches

Although the 2SLS estimator is consistent when the IV assumptions hold 16, 17, inferences may be inefficient because the binary form of *z*_*i*_ is not respected. As an alternative to 2SLS, we can replace (3) with


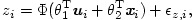
(6)

where Φ(·) is the cdf of the standard normal distribution. The implied (nonlinear) 2SLS procedure fits (6) using nonlinear least squares (NLS) or a generalized linear model, sets 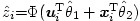, and then evaluates 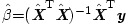. The interpretation of β_1_ is unchanged by the nonlinear first-stage equation because *z*_*i*_ is the main effect in the outcome equation, not 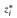.

Following Terza *et al.* 4 we term this approach ‘two-stage predictor substitution (2SPS).’ Despite the fact that orthogonality of 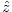 and ε is no longer enforced, 2SPS has been said to yield consistent estimates of β_1_ when the outcome equation is linear and (θ_1_, θ_2_) is estimated consistently 5. However, in the case when the outcome equation is nonlinear, 2SPS has been shown to perform poorly even when the first-stage equation is linear 4.

In the case of a linear model for *z*_*i*_, point estimates of β_1_ under the model


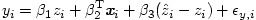
(7)

are identical to those obtained from (4). However, when *z*_*i*_ depends on a nonlinear model such as (6), the effect of *z*_*i*_ above and beyond the effect of 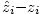 (the ‘endogenous (bad) variation’ in *z*_*i*_) on *y*_*i*_ does not equal the effect of 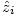 (the ‘exogenous (good) variation’ in *z*_*i*_) on *y*_*i*_. The two-stage residual inclusion (2SRI) procedure of 4, whose origins date to a test for endogeneity in 18, is the estimation of (6) followed by (7). It has been shown that 2SRI yields consistent estimates for linear and nonlinear models 13, Chapter 12.

### 3.4. Maximum likelihood

Assuming normality and using the parameterization of Section 2.2, it follows that


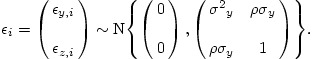
(8)

In this model ρ quantifies the extent to which unobserved factors affecting 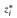 are correlated with those affecting *y*_*i*_ 19. A positive value of ρindicates atypical-favorable selection because unobserved factors that make individuals more likely to take an atypical are also more likely to have higher health costs; i.e. ignoring selection would lead to over estimation of β_1_.

The joint marginal density for (*y*_*i*_, *z*_*i*_) is obtained by integrating over 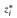 in the model defined by (1), (2), and (8) (see Appendix B for details). The product of these densities is the observed data likelihood function for (β, θ, ρ, σ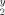), given by


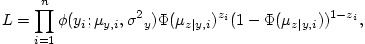


where


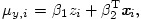
(9)


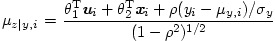
(10)

for ρ≠ ± 1. The presence of µ_*y, i*_ and thus β in (9) and (10) precludes separate maximization of the *y*_*i*_ and *z*_*i*_∣*y*_*i*_ components of the likelihood function. The two components need to be fit simultaneously in order for 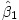 to have a structural shift interpretation. Only when ρ = 0 does it suffice to fit separate linear and probit regression models.

### 3.5. Bayesian inference

Bayesian analysis provides a more flexible approach to inference than maximum likelihood by incorporating prior distributions containing information about the parameters. In the absence of prior information, a non-informative prior is often reasonable.

Because it is the mechanism governing selection, ρ plays a crucial role in the offsets analysis. We are interested in the sensitivity of the results to the prior for ρ and the extent to which it characterizes the other approaches. Prior distributions for ρ that cover a wide range of levels of precision will be used.

Conditional on ρ, the other model parameters are well identified by the data. Therefore, to investigate sensitivity to the prior on ρ, we specify priors diffuse in (β_1_, β_2_, θ_1_, θ_2_, σ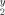) and with varying levels of informativeness about ρ. Specifically, we assume


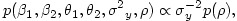


where (ρ + 1)/2∼Beta(ν_1_, ν_2_); *p*(ρ) has the shape of a Beta density but its support is extended from [0, 1] to [−1, 1]. Note that 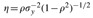 maps the correlation coefficient to (−∞, ∞). In the special case where ν_1_ = ν_2_ = 1, ρ∼U(−1, 1) and *p*(η∣σ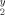) = σ_*y*_{2(1 + σ
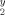
η^2^)^3/2^}^−1^, the density of a *t*-distribution with two degrees of freedom, mean 0, and scale parameter (2σ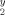)^−1/2^; a thick-tailed distribution.

The values considered for ν = (ν_1_, ν_2_) are such that *E*[ρ] = 0 and 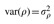. This requires that 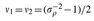, which places a supremum of 1 on 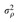 (a bound that is only obtained in the limiting case where *p*(ρ) has point masses of 1/2 at ± 1). The larger ν_1_ = ν_2_ the smaller 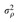.

Although β_1_ is the same conditional effect as for 2SLS and maximum likelihood, Bayesian interpretations are conditioned on data^obs^ = {*y*_*i*_, *z*_*i*_, ***x***_*i*_, ***u***_*i*_}_*i* = 1, …, *n*_.

### 3.6. Testing the exclusion restriction

While the exclusion restriction is a necessary condition for parameter identifiability in the two-stage approaches, the specification of a parametric distribution for ε_*i*_ makes the exclusion restriction non-essential for identifiability of likelihood-based procedures. Therefore, the exclusion restriction may be tested by fitting the model


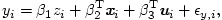
(11)

where ε_*i*_ is specified as in (8). Equation (11) is equivalent to the selection model in 20 and is a special case of the structural shift model in 21. The model is fully identified when ε_*i*_ is bivariate normal if (***x***_*i*_, ***u***_*i*_) contains at least one non-constant predictor 22. A small non-significant value of 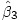 supports the exclusion restriction. However, we emphasize that this test is only valid if ε_*i*_ truly is bivariate normal, an assumption that itself cannot be fully evaluated using the observed data.

### 3.7. Computation

The two-stage procedures can be implemented using standard methods for fitting linear or generalized linear models. However, the computation of standard errors is complicated by the need to simultaneously account for the estimation error from both equations. Equation (5) may be used for 2SLS while asymptotic approximations, such as those outlined in 13, Chapter 12, are needed to obtain closed-form expressions for 2SPS and 2SRI.

Because the likelihood function depends on unobserved latent variables, specialized model-fitting routines are needed for maximum likelihood and Bayesian inferences. MLEs are obtained by directly maximizing the observed data log-likelihood function in (10) using a nonlinear optimization package in R. Standard errors are computed using the delta method to obtain closed-form expressions approximating the covariance matrix of the parameters or functions thereof that is then evaluated at the MLEs of the parameters (see Appendix C). WinBUGS 23 is used for Bayesian inference with inferences evaluated as Monte Carlo averages over draws from the posterior distribution of the model parameters. Convergence is monitored using trace plots and the diagnostics available in CODA 24.

## 4. Cost-offsets: atypical and conventional antipsychotic use in adults with schizophrenia in Florida

The dependent variable is the log-transformed aggregate spending for all services with mental health diagnoses, mental health procedures (e.g. psychotherapy), or psychotropic drugs that are primarily used for mental health treatment such as antidepressants and mood stabilizers for a patient in a given year. The objective is to infer β_1_, the difference in the annual log-spending of treatment of using an atypical versus a conventional antipsychotic for individuals suffering from schizophrenia in Florida's Medicaid population during 1994–2001 when all other factors, including unmeasured factors influencing ε_*i*_, are fixed.

To facilitate interpretation we transform estimates from log-spending to spending (units of $). At a given value of (*z*_*i*_, ***x***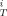)^T^, mean spending equals the exponential of mean log-spending multiplied by a retransformation factor. The retransformation factor may in general be estimated using the smearing estimate 25, given by 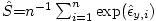, where 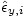 is the estimated residual in (1). Therefore, in $the savings attributed to using atypicals over conventionals is given by


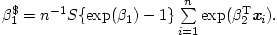
(12)

Under likelihood-based approaches there are alternatives to the smearing estimate. For example, the MLE of the retransformation factor is given by 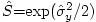. In Bayesian implementations, the retransformation factor from log-normal to normal, *S* = exp(σ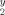/2), is incorporated in the posterior means of any quantities on the scale of the retransformed outcome and so is automatically accounted for when quantities of interest are evaluated as Monte Carlo averages over draws from the posterior distribution of the model parameters.

Another quantity of interest is the average treatment effect (ATE). The ATE evaluates the combined effect of selection and treatment on spending by evaluating the expectation with respect to *f*(*y*_*i*_(*z*)), the marginal distribution of *y*_*i*_(*z*) after integrating over 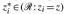, whereas β_1_ is the pure effect of the latter. The average treatment effect over individuals with covariates values 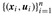 is given by


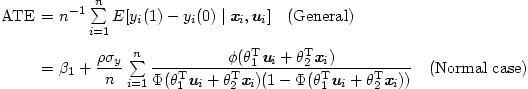
(13)

or in terms of dollars


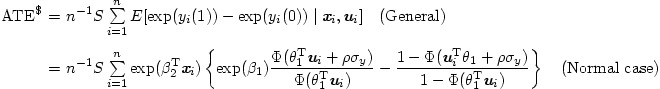
(14)

where ϕ(·) and Φ(·) denote the pdf and cdf of the standard normal distribution, respectively. Equation (13) illustrates that ATE = β_1_ when ρ = 0 (no selection).

A feature of parametric methods is that they are able to delineate between population effects (e.g. β_1_) and (average) treatment effects specific to the subjects in the sample. The expressions for the ATE's in (13) and (14) are informative because they show their relationship to β_1_. Such expressions can only be determined through the specification of a full parametric model for the data.

The local average treatment effect (LATE), the effect of treatment on those whose treatment status can be changed by ***u***_*i*_ (the marginal population), equals β_1_ when there is a single binary instrument, no covariates, the exclusion restriction holds, and the effect of ***u***_*i*_ is monotone across *i* 26, p. 155. However, under our model different LATEs correspond to the values of ***u***_*i*_ defining the marginal population. Specified mathematically, the LATE is given by *E*[*y*_*i*_(1) − *y*_*i*_(0)∣*z*_*i*_(***u***^(1)^)>*z*_*i*_(***u***^(0)^), ***x***_*i*_], where *z*_*i*_(***u***^(*k*)^) is the potential treatment of subject *i* when ***u***_*i*_ = ***u***^(*k*)^ (*k* = 0, 1). (See 27, 28 for summary measures of heterogeneous LATE.) However, β_1_ (the 2SLS estimand) can be thought of as a weighted average of a LATE for the marginal subpopulations identified (one at a time) by each component of ***u***_*i*_. Therefore, despite not corresponding to a single LATE, our primary interest is in β_1_ and so we do not report LATE for any particular subpopulations.

To gauge the sensitivity of results computed under the Bayesian model with respect to the prior for ρ, we fit this model with 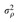 between 0.96 (prior has a U-shape) and 10^−4^/3 (prior is a spike at 0).

### 4.1. Descriptive results

The Florida Medicaid data set comprises 26 759 adults diagnosed with schizophrenia at some point during 1994–2001 yielding *n* = 78349 person-year observations (Table I) of health care spending. The vector ***x***_*i*_ has 18 elements (intercept, black, other non-white (largely Latino), female, age, receipt of supplemental security income (SSI), substance abuse history, year, and 10 area dummies), whereas ***u***_*i*_ contains 33 elements (the availability of zyprexa, seroquel, and geodon and their interactions with area).

**Table I:** Florida Medicaid spending for atypical users compared to conventional users (78 378 person-year observations)

		Mental Health (MH) Spending ($) (Mean, StDev)	
		
Variable	Mean	Atypical	Conventional
Atypical	0.450	11 713 (12 083)	
Conventional	0.550		6218 (8833)
Male	0.476	12 034 (12 190)	6754 (9206)
Female	0.524	11 425 (11 980)	5727 (8445)
White	0.430	12 283 (11 998)	6365 (8759)
Black	0.266	11 656 (11 996)	6235 (8833)
Other non-white	0.304	10 994 (12 221)	5994 (8931)
Substance abuse	0.120	21 241 (14 844)	14 694 (13 070)
Non-substance abuse	0.880	10 103 (10 748)	5270 (7662)
SSI	0.964	11 872 (12 199)	6291 (8891)
non-SSI	0.036	7647 (7565)	4146 (6646)
Zyprexa available	0.730	11 420 (11 896)	5737 (8277)
Zyprexa not available	0.270	13 931 (13 207)	6953 (9573)
Seroquel available	0.589	11 315 (11 816)	5533 (7986)
Seroquel not available	0.411	13 076 (12 867)	6750 (9403)
Geodon available	0.159	11 999 (12 717)	6001 (8817)
Geodon not available	0.841	11 619 (11 867)	6239 (8834)
Pensacola	0.041	7323 (10 150)	6475 (12 660)
Tallahassee, Panama City	0.045	6991 (10 012)	5220 (10 368)
Gainesville, Ocala	0.065	6209 (9568)	4436 (9430)
Jacksonville, Daytona Beach	0.099	833 (11 172)	6664 (11 976)
St. Petersburg	0.069	8077 (10 654)	6081 (11 611)
Tampa, Lakeland, Bradenton	0.049	6861 (8855)	4365 (7268)
Orlando	0.072	7775 (11 707)	5552 (11 006)
Ft. Myers, Sarasota, Naples	0.031	6639 (9671)	4434 (8960)
West Palm Beach, Vero Beach	0.058	7517 (11 080)	5685 (12 130)
Ft. Lauderdale	0.086	9768 (13 092)	7886 (13 865)
Miami, Key West	0.384	10 154 (13 445)	6807 (12 349)

		Correlation with MH spending	
		
Variable	Mean (SD) or range	Atypical	Conventional
Year	1994–2001	−0.0425	−0.0655
Age	42.57 (11.37)	−0.0374	−0.0791

A comparison of means based on Table I suggests that atypical antipsychotics are much more expensive than conventional drugs. However, this analysis does not account for non-random selection of patients into treatment; for example, patients with more severe conditions may have higher propensity to receive an atypical and also be more costly. It is clear from Table I that spending is higher for males, whites over blacks, blacks over other non-whites, substance abusers, and those receiving SSI. For all predictors other than male (versus female), the magnitude of the difference is greater within atypical antipsychotics than conventionals. However, the magnitude of the correlations between year and spending, and between age and spending, was greater for conventional antipsychotics.

Because the distribution of cost _*i*_ is naturally skewed to the right, we sought a transformation that induced normality. The maximum likelihood estimate of the Box–Cox transformation is 0.140 for unadjusted cost_*i*_, 0.153 under OLS, and approximately − 0.05 under the fully parametric simultaneous equations model. Because the log-transformation is a compromise between these alternatives, corresponding to a transformation parameter of 0, we transformed the data using *y*_*i*_ = log(cost_*i*_). Using the alternative Box–Cox transformations had minimal effect on the results of the analysis.

### 4.2. Treatment effects

We analyzed the data using each of the procedures discussed in Section 3: ordinary least squares (OLS), two-stage least squares (2SLS), two-stage predictor substitution (2SPS), two-stage residual inclusion (2SRI), maximum likelihood, and Bayesian analysis with various priors.

**Figure 1:**
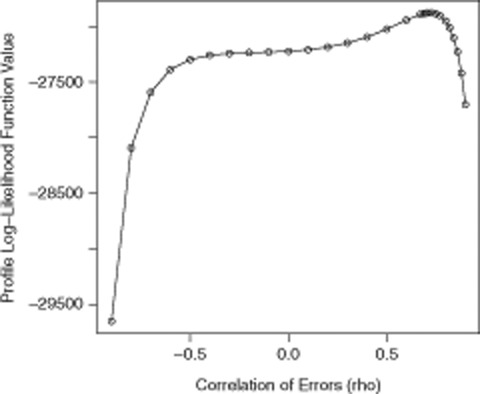
Profile likelihood of ρ based on 78 378 observations from the Florida Medicaid data set.

The large value of ρ estimated by the likelihood-based methods (MLE and Bayesian models) was verified by plotting the profile likelihood function of ρ and confirming the existence of a unique global optimum at 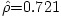, far from the edge of the parameter space (Figure 1). Under such a strong unmeasured selection effect, β_1_ and ATE are destined to have very different values. The Staiger–Stock test *F*-statistic of 9.86 in the 2SLS analysis suggests that the instrument only accounts for a small fraction of the selection effect and would be considered borderline-weak compared to the conventional standard of 10 29.

Ordinary least squares (OLS) suggests that the newer atypical antipsychotics result in more spending (Table II: β_1_ estimated to be near 1), the two-stage procedures give inconclusive results (β_1_ estimated to be near 0), and the likelihood-based methods suggest that the newer atypicals lead to lower levels of spending (β_1_ estimated to be near − 0.7). In terms of annual patient dollars, the cost of atypicals less the cost of conventionals was estimated to be $9948, range from − $263.3 to $2262, and range from − $10010 to − $9065 under OLS, the two-stage procedures, and the likelihood-based procedures, respectively. Because the left-skewness of the data inflates the treatment effect upon retransformation, the predicted mean OLS estimate is substantially larger than the raw mean difference (Table I). Inflation of the mean due to retransformation combined with the highly positive selection effect leads to the large saving found under the likelihood-based analyses.

**Table II:** Point estimates of the treatment effects (and associated uncertainty) for the ordinary least squares (OLS), two-stage least squares (2SLS), two-stage predictor substitution (2SPS), two-stage residual inclusion (2SRI), maximum likelihood (MLE), and the Bayesian procedures on the Florida Medicaid population

		Two-stage				Likelihood-based	
			
Term	Quantity	OLS	2SLS	2SPS	2SRI	MLE	Bayesian
β_1_	Estimate	1.022	−0.028	0.237	0.193	−0.793	−0.773
	Standard deviation	0.010	0.169	0.144	0.145	0.031	0.031
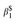	Estimate	9 965	−263.3	2 262	1 911	−9393	−9948
	Standard deviation	122.8	1 607	1 415	1 668	537.2	531.5
ATE	Estimate					1.049	1.013
	Standard deviation					0.010	0.010
ATE ^$^	Estimate					9 576	10 190
	Standard deviation					107.1	94.3
ρ	Estimate					0.721	0.696
	Standard deviation					0.008	0.008

The ATE, ATE ^$^, and ρ are only estimated for the likelihood-based procedures as estimation relies on the specification of a probability distribution for the observations.

The OLS estimate of β_1_ and the likelihood-based estimate of the ATE are fairly similar, illustrating that the former is actually estimating the ATE. The SE of the MLE of the ATE is slightly smaller than the SE of the OLS estimate, consistent with the result in 30 that regression parameters of terms unique to one regression equation are estimated more efficiently in a bivariate model than with the corresponding univariate model.

Despite estimating the same quantity, differences between the two-stage and likelihood-based estimates of β_1_ are substantial. Because we thoroughly check the distribution of the observed variables graphically and using several diagnostics, and also explored various variable transformations, we believe that the discrepancy in these estimates is due to things we do not observed that cannot be tested fully empirically: violations of the exclusion restriction or departures of the distribution of the error terms from the bivariate normal distribution of the data. To gain further insight into possible causes of the discrepancy we conducted a simulation study (Section 5).

The 95 per cent confidence interval for β_1_ under 2SLS only just overlaps 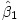 under 2SPS and 2SRI and conversely the 95 per cent confidence intervals of β_1_ under 2SPS and 2SRI only just encompass 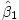 under 2SLS, illustrating that the results are sensitive to small differences in the method of estimation. The MLEs had SEs about one-fifth and one-third those for 2SLS and its nonlinear variants (2SPS and 2SRI), respectively, thus highlighting the ability of the likelihood procedures to yield more precise inferences.

**Figure 2:**
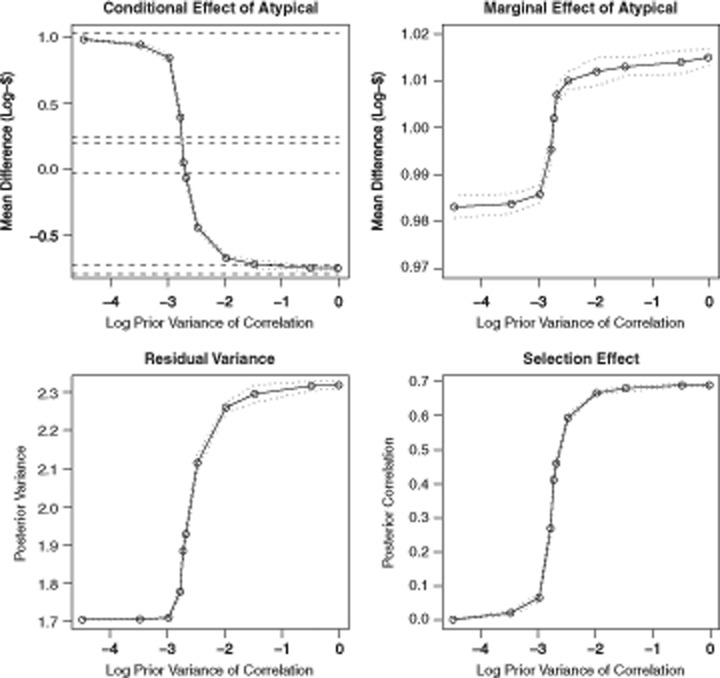
Relationship between Bayesian posterior means of (β_1_, ATE, σ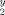, ρ) and log_10_ of the prior variance, 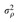, when ρ has an extended Beta density on [−1, 1]. The dotted lines are the interpolated pointwise 99 per cent credibility intervals. The dashed lines in the upper left-hand plot depict from top to bottom the OLS, 2SPS, 2SRI, 2SLS, Bayesian posterior mean under a U(−1, 1) prior for ρ, and the MLE, respectively.

Comparing the scale of the vertical axes in Figure 2, the Bayesian point and interval estimates of β_1_ and 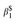 were substantially more sensitive to *p*(ρ) than Bayesian estimators of ATE and ATE ^$^. From 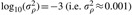 to 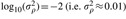, *E*[β_1_∣data^obs^] (and thus 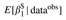) move from being close to the OLS estimate to close to the MLE. However, as indicated in the plot of the selection effect (bottom-right), a very precise prior on 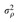 is required to obtain Bayesian estimates that correspond to those of the two-stage approaches.

The 33 elements of ***u***_*i*_ in (11), the model for testing the exclusion restriction, had standardized effects (estimate divided by standard error or posterior standard deviation) ranging from 1.054 to 1.954, not significant at the 0.05 level. The *F*-statistic for the test that β_3_ = 0 equals 237, well above the critical value at the 0.05-level of 47.4. Thus, there is strong evidence under the assumed bivariate normal model that the exclusion restriction is violated.

## 5. Simulation study

We conducted a simulation study to evaluate the sensitivity of the estimators of β_1_ and the ATE, and properties of likelihood-based tests of the exclusion restriction (i.e. the condition β_3_ = 0), to the distribution of ε_*i*_. Computations were streamlined by substituting ***x***_*i*_ and ***u***_*i*_ with the univariate variables *x*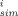 and *u*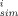, whose effects approximate the combined effects of all elements of ***x***_*i*_ and ***u***_*i*_, respectively. This was achieved by making the variance of *x*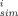 and *u*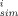 equal 1 and β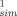, θ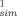 and θ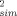 equal the empirical standard deviation of β
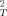
***x***_*i*_, θ
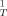
***u***_*i*_ and θ
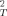
***x***_*i*_, respectively. To further reduce computation time while emulating the Florida Medicaid data, both *n* and σ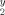 were reduced by factors of 10. Bias, mean-squared error (MSE), and coverage were estimated by averaging over 1000 simulated data sets.

In the first group of simulations, ε_*i*_ was drawn from a bivariate normal distribution, the case where the likelihood function is correctly specified. In subsequent simulations, observations were randomly drawn from a bivariate *t*-distribution with seven degrees of freedom or were correlated draws from gamma distributions, allowing assessment of the robustness of the approaches to thicker-tailed and skewed distributions. Finally, we simulated data in violation of the exclusion restriction by setting β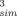 = θ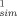 to evaluate sensitivity with respect to the exclusion restriction. We also evaluated the normal likelihood-based test of the exclusion restriction for β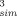 ranging from 0 to θ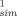.

**Figure 3:**
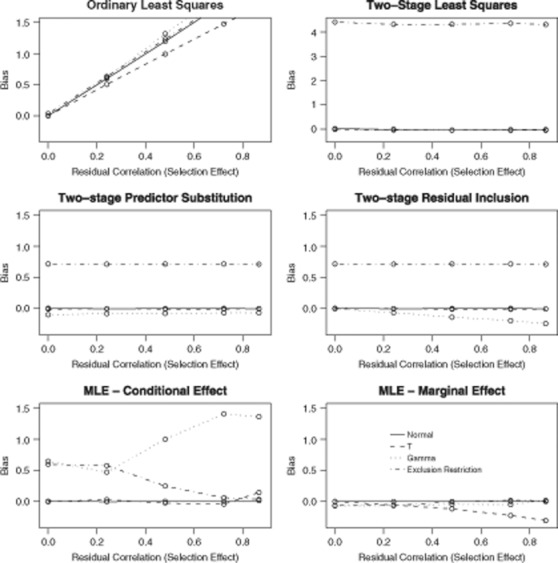
Simulated bias of estimators as a function of ρ for different outcome distributions and status of the exclusion restriction. As per the Florida Medicaid analysis β_1_ = − 0.793, θ_1_ = 0.144, and if the exclusion restriction is violated then β_3_ = 0.144. The vertical axis in the upper-right plot covers a wider range to accommodate the excessive bias of 2SLS under violation of the exclusion restriction.

**Figure 4:**
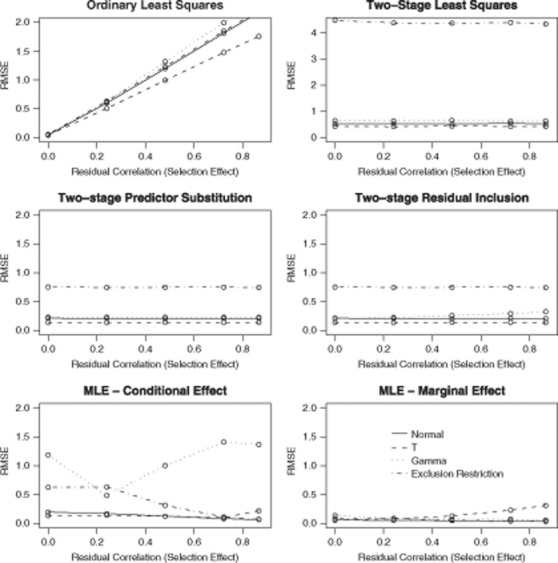
Simulated root mean-squared error (RMSE) of estimators as a function of ρ for different outcome distributions and status of the exclusion restriction. As per the Florida Medicaid analysis β_1_ = − 0.793, θ_1_ = 0.144, and if the exclusion restriction is violated then β_3_ = 0.144. The vertical axis in the upper-right plot covers a wider range to accommodate the excessive RMSE of 2SLS under violation of the exclusion restriction.

**Figure 5:**
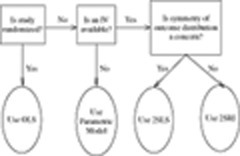
Algorithm for choosing the best method in practice. The decision process begins with the left-hand rectangle and at each subsequent step selects a method (ovals) or moves to the next decision (boxes). Because our results suggest that 2SPS is dominated by either 2SRI or 2SLS, it does not appear. Data transformations and other analyses that inform model specification should be performed prior invoking this algorithm.

The bias and root mean square error (RMSE) for each estimator and scenario are displayed in Figures 3 and 4, respectively, while Table III contains operating characteristics of the likelihood-based test of the exclusion restriction. However, in discussing these results we use a method-by-method approach; this was most helpful in describing the scenarios under which each approach works best and when it absolutely should not be used. To supplement the results, Figure 5 depicts an algorithm for determining which approach is best to use in practice.

**Table III:** Simulations results when the exclusion restriction may be violated (ρ = 0.721)

	Parameter values			Statistics				
		
Distribution	β_1_	θ_1_	β_3_	Bias	RMSE	Coverage	*z*-Value	Power
Normal	−0.793	0.144	0.000	0.000	0.018	0.968	−0.025	0.054
Normal	−0.793	0.144	0.036	0.001	0.018	0.972	2.055	0.526
Normal	−0.793	0.144	0.072	0.000	0.017	0.984	4.077	0.982
Normal	−0.793	0.144	0.108	0.000	0.017	0.972	6.060	1.000
Normal	−0.793	0.144	0.144	0.000	0.018	0.980	8.075	1.000
*T*	−0.793	0.144	0.000	0.001	0.015	0.974	0.054	0.038
*T*	−0.793	0.144	0.036	0.001	0.015	0.970	2.470	0.707
*T*	−0.793	0.144	0.072	0.001	0.015	0.982	4.802	0.996
*T*	−0.793	0.144	0.108	0.003	0.016	0.946	7.322	1.000
*T*	−0.793	0.144	0.144	0.002	0.015	0.972	9.651	1.000
Gamma	−0.793	0.144	0.000	−0.043	0.046	1.000	−2.767	0.802
Gamma	−0.793	0.144	0.036	−0.044	0.046	1.000	−0.498	0.066
Gamma	−0.793	0.144	0.072	−0.045	0.047	1.000	1.723	0.390
Gamma	−0.793	0.144	0.108	−0.044	0.047	1.000	4.054	0.988
Gamma	−0.793	0.144	0.144	−0.043	0.046	1.000	6.389	1.000

### 5.1. Results for OLS

As expected the OLS estimates became increasingly biased the further ρ was from 0 (Figure 3) with RMSE is essentially equal to bias in all cases where ρ≠0. Any variations in its performance across distributions or under violation of the exclusion restriction (which is irrelevant as far as OLS is concerned) were drowned out by the impact of an unmeasured confounder. Clearly, if there are legitimate concerns about unmeasured confounders then OLS is not appropriate.

### 5.2. Results for 2SLS

Figure 3 shows that 2SLS performs well across all conditions other than violation of the exclusion restriction, in which case 2SLS is even more biased than OLS and thus should not be used. However, if the IV is supported by theoretical arguments or other insights indicating that the exclusion restriction holds use of 2SLS is appropriate (as shown in Figure 5).

It is clear from Figure 4, where the range of the vertical axis for 2SLS is much greater than that of the other methods, that the standard errors of 2SLS estimates exceed those of all other methods. Thus use of 2SLS typically lowers the statistical power of the analysis compared to other approaches.

### 5.3. Results for 2SPS and 2SRI

As shown in Figures 3 and 4 these alternative moment-based IV procedures compared favorably to 2SLS when the underlying distribution is symmetric or the exclusion restriction is violated (although they still perform consistently poorly in this scenario) but not so favorably when the underlying distribution is skewed. In general, 2SRI is more precise (smaller variance and RMSE) than 2SPS which is more precise than 2SLS while the reverse order holds for sensitivity to skewness (i.e. 2SRI is worst performed).

These results, not previously reported in the literature, may be a consequence of the nonlinearity of Equation (7) introducing bias when the distribution of ε_*i*_ is skewed. Because the theoretical results reported in 4 imply that 2SRI and 2SPS are consistent (irrespective of the underlying distribution), bias should approach 0 as *n* increases. However, additional simulations at different values of *n* suggested that, at best, the convergence is very slow.

Based on the above, 2SRI may be the best method to use when the evidence supporting the validity of the IV is strong (as for 2SLS) but *n* is such that the study is insufficiently powered under 2SLS. However, if there is evidence that ε_*i*_ has a skewed distribution, particularly ε_*y, i*_ (see Section 5.5), then 2SLS would be the safer (more robust) choice.

### 5.4. Results for likelihood-based estimators

The MLE and the Bayesian estimators of β_1_ yield better results than the two-stage estimators when the underlying distribution is normal and are more robust to violations of the exclusion restriction. However, they are more sensitive to departures of the underlying distribution from normality. An interesting finding is that likelihood-based estimators of β_1_ are relatively more robust when non-normality is in the form of thicker tails than skewness while the reverse is true for likelihood-based estimators of the ATE.

The robustness of the MLE of the ATE is due to the presence of ρ. The two-stage methods do not account for ρ and so, with no way to compensate for β_3_≠0, yield biased results, while the MLE of β_1_ is only partially affected by violations of the exclusion restriction due to the fact that the error correlation ρ partially absorbs β_3_*u*
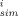
≠0.

As indicated in Figure 5, likelihood-based methods are recommended when unmeasured confounders are thought to exist but it is questionable whether the IV is valid or no IV is available. To make likelihood-based analyses as believable as possible, transformations of *y*_*i*_ that induce normality in 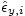 should be considered.

#### 5.4.1. Test of exclusion restriction

Table III shows the results of including *u*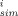 as a predictor of *y*_*i*_ when the true value of its coefficient, β_3_, varies from 0 to 0.144 (i.e. up to the magnitude of the effect of the IV). When the underlying distribution is bivariate normal, β_3_ is estimated with high precision and no bias. Furthermore, the power of the test *H*0: β_3_ = 0 against the alternative *HA*: β_3_≠0 increases from 0.05 when the true value is 0 (in this case power = type I error) to over 0.95 at 0.072. Therefore, if normality holds the likelihood-based methods provide a valid test of the exclusion restriction.

Inferences about β_3_ are almost as reliable if the underlying distribution has *t*_7_ as opposed to normal marginals, slightly over-covering when β_3_>0. However, if the underlying distribution is skewed (as for a Gamma distribution), then estimates of β_3_ are biased and the type I error of the test of the exclusion restriction is excessive. Therefore, the bivariate normal test of the exclusion restriction cannot be relied upon if the true outcome distribution is asymmetric.

### 5.5. Other results

We also evaluated the approaches under various other scenarios for which we do not present results. In one series of simulations one of ε_*y, i*_ and ε_*z, i*_ was normal and the other was non-normal (*t* or gamma) distributed. Results were more sensitivity to non-normality of ε_*y, i*_ than ε_*z, i*_. In fact, as long as ε_*y, i*_ was symmetric, 2SRI appeared to be robust to the distribution of ε_*z, i*_ and the likelihood-based procedures were only biased by small amounts.

When θ_1_ increases by a factor of 2, the MSE of the two-stage estimators decreases by a factor of 4. Although the MLE becomes more precise as θ_1_ increases, the trend is nowhere as dramatic as for the two-stage approaches. This reflects the fact that the likelihood-based procedures are identified from the distribution of the data and so the involvement of *u*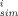 improves the stability and precision of the estimates. Multiplying β_1_ by 2 does not alter the precision of the estimators revealing that the magnitude of β_1_ is not tied to the precision with which it is estimated.

The performance of interval estimators for β_1_ was highly correlated with the bias and variance of the corresponding point estimators. If the point estimator was unbiased then the coverage of the interval estimator was close to 0.95.

## 6. Discussion

We used data from a large state database to investigate whether newer atypical antipsychotics lowered net costs of health care relative to conventional antipsychotics. Because treatment is non-randomly assigned, instrumental variables methods were used to separate the true effect of treatment on log-cost from selection effects. To aid interpretation, we converted the total payments made under each treatment from log-spending to spending (in $). We used several approaches for the analysis with the rationale that the methods would validate one another if similar results were obtained. The methods yielded results that were surprisingly disparate; atypicals were estimated to save about $10 000 under likelihood-based procedures (the MLE and Bayesian models), in contrast to no saving or increased spending, of about $2000, under the two-stage procedures. These results bring the assumptions underpinning the methods into question.

To gain a sense of which results to believe, we used simulations that studied the properties of the two-stage procedures and the MLE. We observed the following: (1) OLS only works in the absence of confounding, (2) 2SLS works well in all scenarios other than when the exclusion restriction is violated, in which case it fails completely, (3) 2SPS performs better than 2SLS unless the underlying distribution is skewed, (4) 2SRI performs better than 2SPS unless the underlying distribution is skewed, and (5) likelihood-based estimators perform better when the underlying distribution is normal and when the exclusion restriction is violated. While results (1) and (2) are well known, (3)–(5) are new findings. The poor performance of the alternative two-stage procedures when the distribution of the data is asymmetric illustrates that the complete robustness of 2SLS to the distribution of the data is compromised by seeking to improve efficiency through the use of a nonlinear first-stage equation.

Likelihood-based estimators of β_1_ and β_3_ (the direct effect of the candidate IVs on the outcome) are surprisingly robust to violations of normality as long as the true distribution is symmetric, but fail when the true distribution is skewed. Thus, while likelihood-based models can be robust and, therefore, can be used to test the validity of 2SLS when the true distribution is symmetric, their findings are quickly compromised if the true distribution is skewed.

With the above in mind, where does the evidence for offsets of atypical antipsychotics point? Based on the analysis of the Florida Medicaid data, there is evidence that the assumption of normal residuals, although a reasonable approximation, does not hold exactly. Therefore, because 2SLS is robust to the distribution of the residuals and the exclusion restriction can be defended heuristically (i.e. from an economic standpoint), 2SLS might be most trustworthy. However, the findings in this paper reveal that even a small departure from the exclusion restriction makes 2SLS and the alternative two-stage procedures likely to produce results that are substantially biased. Given the highly conflicting results across the approaches we feel there is insufficient evidence to conclude the offset of atypical antipsychotics is positive or negative. This is generally consistent with the research from clinical trials such as the Clinical Antipsychotic Trials of Intervention Effectiveness (CATIE) study 31.

The fact that different statistical procedures result in such different results is alarming. The finding that the alternative two-stage procedures are sensitive to the underlying distribution of the data is important for researchers using these methods. Similarly, the results on the sensitivity of parametric models derived from latent variable constructs, as for the likelihood-based analysis here, is an important lesson to statisticians and other users of these approaches.

The methodology we have developed is generally applicable to any observational study in which an IV is available for the treatment and outcome of interest. In light of the sensitivity results reported here, the availability of a valid IV is critical. However, finding IVs in practice can seem an art form to one not familiar with IV analysis as the arguments supporting the exclusion restriction are theoretically rather than empirically driven. Therefore, we recommend a subject matter expert with an acute sense of the outcome, treatment and unobserved confounding variables is integrally involved in the determination of candidate IVs.

A limitation of our empirical analysis is that we did not account for repeated measurement of subjects that appeared in the data set in multiple years. We subsequently fit a hierarchical Bayesian model that included a random intercept for subject. The posterior mean of β_1_ was − 0.492 (sd = 0.0197), suggesting that single-level analyses might over-estimate the magnitude of β_1_. The within-subject variance had a posterior mean of 1.036 (0.0125) while the between-subject variance had a posterior mean of 1.118 (0.0141), suggesting there is substantial unexplained variance between subjects.

Another direction in which the likelihood-based estimators could be extended is by assuming a more flexible family of distributions for ε_*i*_ (e.g. bivariate *t*-distribution) and constructing estimators under those less restrictive assumptions. However, because a more flexible model is likely to be less well identified by the data, it is not clear that it would yield an estimator that is more robust to the distribution of the data.

## Appendix A: Method-of-moments derivation of 2SLS

Classic IV fits the model in Equation (1) subject to the constraint that ***u***_*i*_ and ε_*i*_ are orthogonal. Write ε_*y, i*_(β) = *y*_*i*_ − β_1_*z*_*i*_ + β***x***_*i*_ to emphasize the dependence of ε_*y, i*_ on β = (β_1_, β_2_). By definition, ***u***_*i*_ is uncorrelated with ε_*y, i*_ and so *E*[***u***_*i*_ε_*y, i*_(β)] = **0** for all *i*. As we do not observe the true expectations, we seek values of β such that

(A)1

Because ***x***_*i*_ is exogenous, *E*[***x***_*i*_ε_*y, i*_(β)] = **0**.

Equation (A1) can be solved exactly if the number of IVs equals the number of endogenous predictors. However, if dim(***u***_*i*_)>dim(*z*_*i*_) it is generally not possible to satisfy all of the orthogonality conditions simultaneously; *z*_*i*_ is said to be overidentified. An ‘optimal’ solution is obtained by finding the parameter values that minimize the quadratic form

where ***W*** is a positive-definite weighting matrix quantifying the relative importance of the orthogonality conditions across the instruments. Setting ***W*** proportional to cov (***X***ε_***y***_) = σ***X***^T^***X***, where ε_***y***_ = (ε_*y*, 1_, …, ε_*y, n*_)^T^, minimizes the variance of *Q*(β). The IV estimator is then the value of β which minimizes *T*(β)^T^***X***^T^***X****T*(β) subject to (A1), yielding

(A2)

where  is the predicted value of ***X*** in a regression of ***X*** on ***U*** and ***U*** is the matrix with *i*th row (***u***, ***x***) 15, p. 530. Because ***x***_*i*_ is contained in both ***X***and ***U***it projects onto itself and so . Geometrically,  is the projection of ***z*** = (*z*_1_, …, *z*_*n*_)^T^ onto ***U***;  is orthogonal to ε_***y***_ and thus contains only the component of variation in ***z*** that can be used to estimate β.

## Appendix B: Derivation of the likelihood function

Make ε_*y, i*_ and ε_*z, i*_ the subject of Equations (1) and (2) and factor the joint density as *f*(ε_*y, i*_, ε_*z, i*_) = *f*(ε_*y, i*_)*f*(ε_*z, i*_∣ε_*y, i*_). Then integrate with respect to ε_*z, i*_ over those values of ε_*z, i*_ for which *z*_*i*_ = 1, and analogously for those values for which *z*_*i*_ = 0, to obtain *f*(*z*_*i*_∣ε_*y, i*_). Finally, substitute for ε_*y, i*_ to obtain the joint density of (*y*_*i*_, *z*_*i*_).

## Appendix C: Computation of standard errors of MLEs

Let ***d***_*i*_ = (*z*_*i*_, ***u***)^T^, θ←θ/(1 − ρ^2^)^1/2^, η = ρ/{σ(1 − ρ^2^)}^1/2^, *r*_*i*_ = *y*_*i*_ − ***d***β and *m*_*i*_ = ***u***θ + η*r*_*i*_. Then set Φ_1_(*m*_*i*_, *z*_*i*_) = {*z*_*i*_/Φ(*m*_*i*_) − (1 − *z*_*i*_)/(1 − Φ(*m*_*i*_))}ϕ(*m*_*i*_), Φ_2_(*m*_*i*_, *z*_*i*_) = {*z*_*i*_/Φ(*m*_*i*_)^2^ + (1 − *z*_*i*_)/(1 − Φ(*m*_*i*_)^2^)}ϕ(*m*_*i*_)^2^, Φ_3_(*m*_*i*_, *z*_*i*_) = − *m*_*i*_Φ_1_(*m*_*i*_, *z*_*i*_) − Φ_2_(*m*_*i*_, *z*_*i*_), and *w*_*i*_ = 1/σ − η^2^Φ_3_(*m*_*i*_, *z*_*i*_).

The first and second derivatives of the log-likelihood function, ℒ, are

All other elements of the Hessian matrix, the matrix of second derivatives of the log-likelihood function with respect to (β, θ, η, σ), equal 0. The covariance matrix of the estimated parameters is estimated by the negative-inverse-Hessian matrix. The approximate variance of estimators of functions of these parameters, such as ρ and the ATE, are then derived using the delta method.
